# Supplementary material for: Genome-wide CRISPR interference screen identifies long non-coding RNA loci required for differentiation and pluripotency
Source: PLoS One. 2021 Nov 3;16(11):e0252848. doi: 10.1371/journal.pone.0252848 (PMC8565776; doi:10.1371/journal.pone.0252848)
Supplement: S1 Raw images — (PDF) [file pone.0252848.s009.pdf]

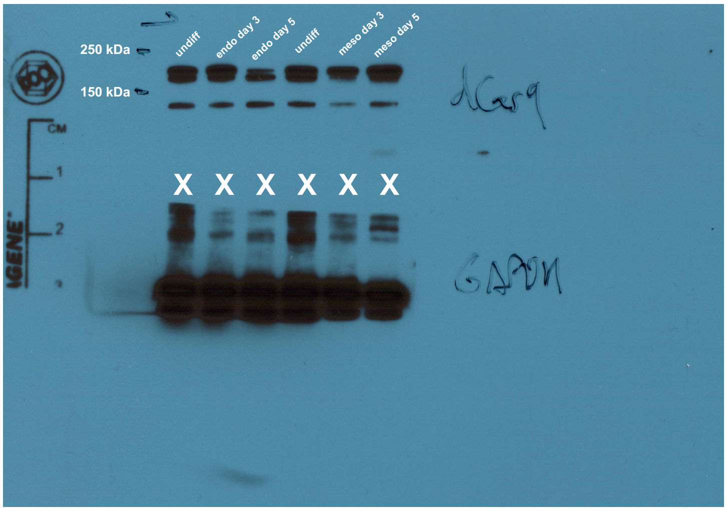

Developed film
dCas9-KRAB
Fig 1B

Developed film
GAPDH
Fig 1B

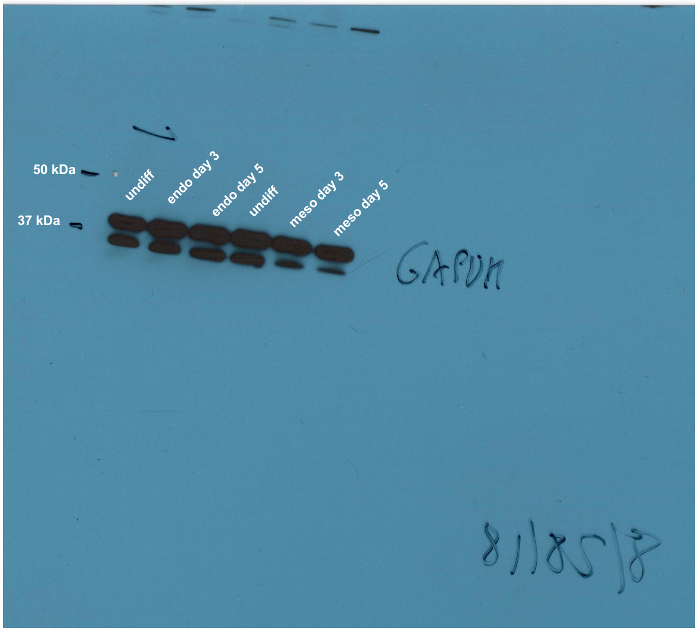

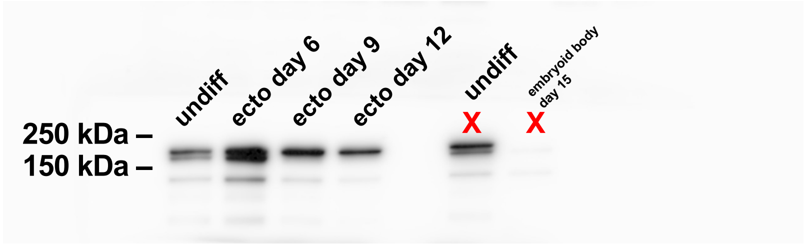

Developed film
dCas9-KRAB
Fig 1C

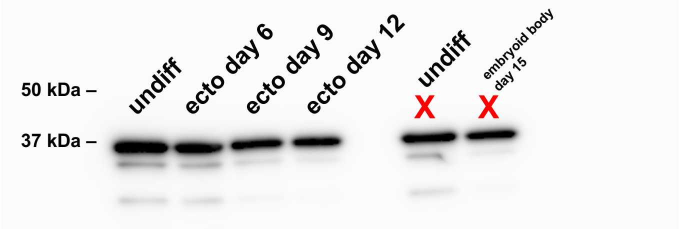

Developed film
GAPDH
Fig 1C

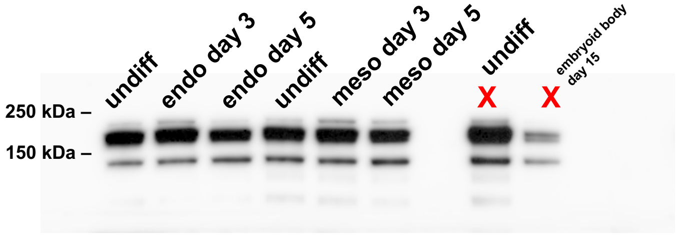

Developed film
dCas9-VP64
Fig S1B

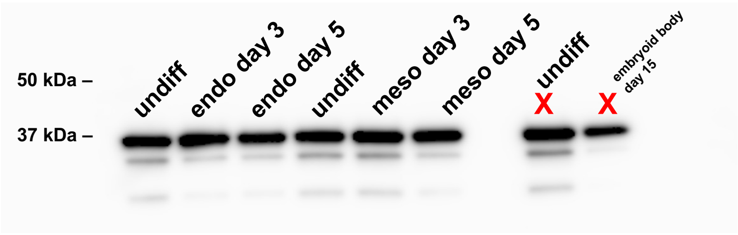

Developed film
GAPDH
Fig S1B

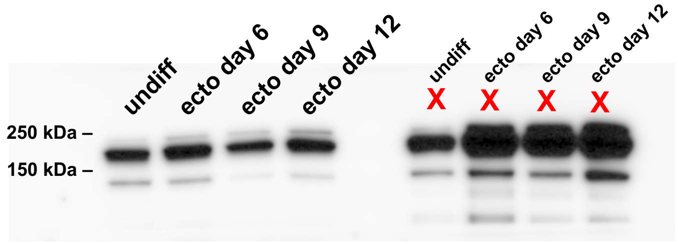

Developed film
dCas9-VP64
Fig S1C

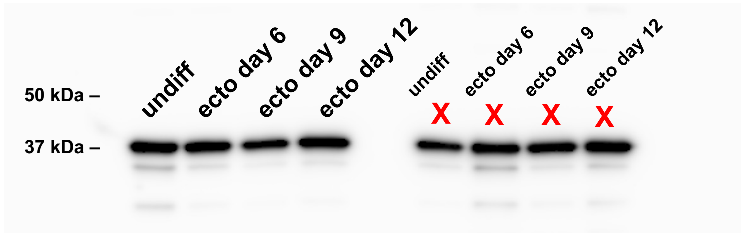

Developed film
GAPDH
Fig S1C

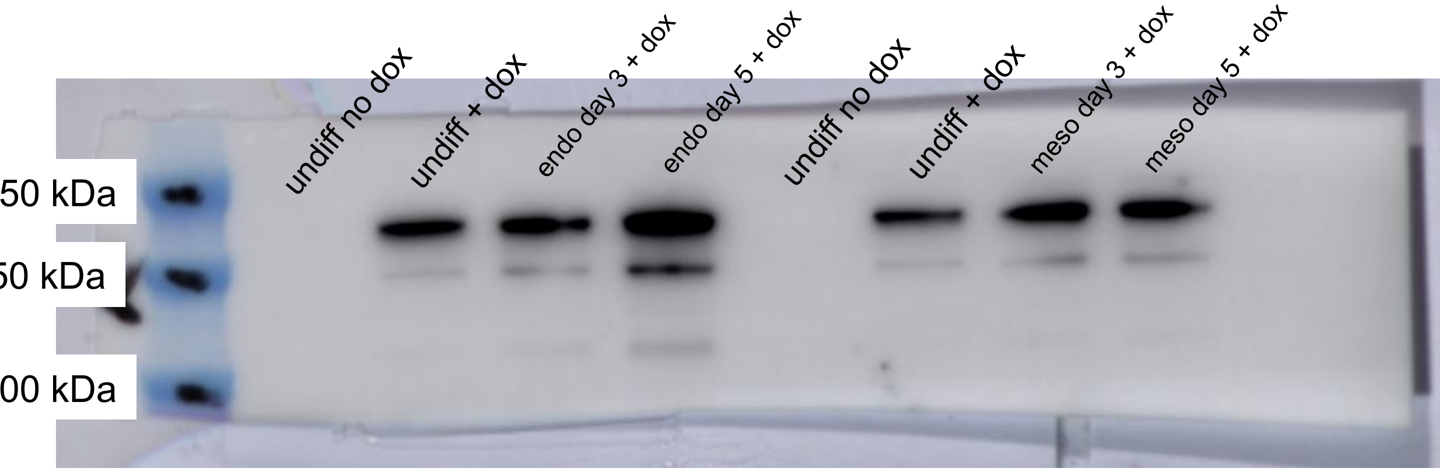

Developed film
dCas9-KRAB
Fig S2B

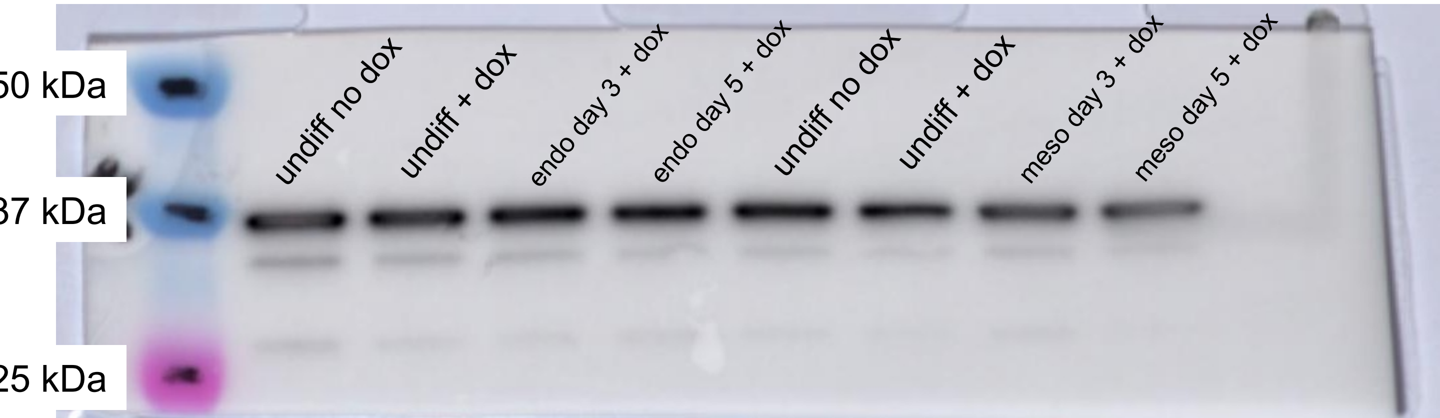

Developed film
GAPDH
Fig S2B

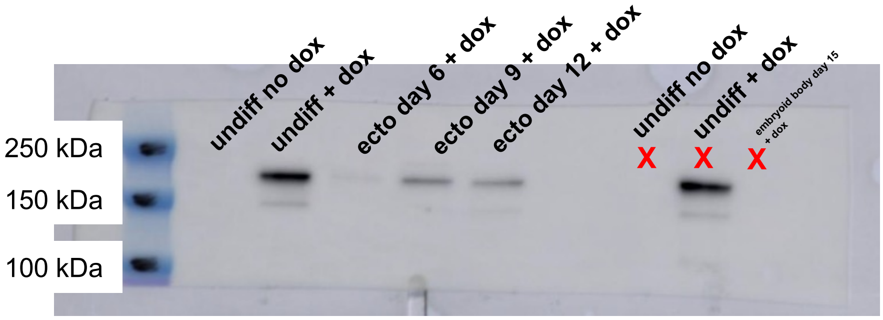

Developed film
dCas9-KRAB
Fig S2C

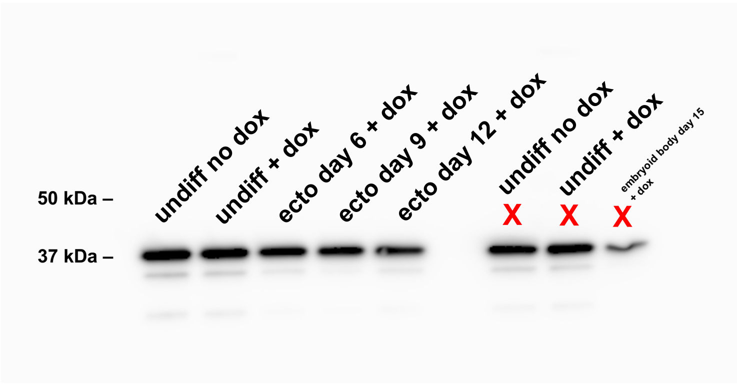

Developed film
GAPDH
Fig S2C
